# Advances in Welding Process and Materials

**DOI:** 10.3390/ma18214904

**Published:** 2025-10-27

**Authors:** Cosmin Codrean, Carmen Opriș, Anamaria Feier

**Affiliations:** Department of Materials and Manufacturing Engineering, Politehnica University of Timisoara, 300006 Timisoara, Romania; carmen.opris@upt.ro (C.O.); anamaria.feier@upt.ro (A.F.)

This editorial aims to highlight current trends in conventional and advanced materials-joining methods, as well as the current challenges in this field. It particularly focuses on the results published in “Advances in Welding Process and Materials (2nd Edition)” to promote this volume among specialists working both in research and in the production of goods.

The exponential development of goods production generates a shortage of raw materials and energy sources, as well as increasing human aggression towards the environment. In this context, an increasing emphasis is placed on protecting the environment and conserving material and human resources through the development and creation of new materials and manufacturing technologies.

As materials become increasingly sophisticated in their chemical composition to provide exceptional performance properties, a more complete and accurate understanding of how such materials can be joined to produce quality products becomes a necessity. Demands to improve productivity, efficiency, and quality represent challenges for welding processes in all industries. Traditional welding technologies will certainly evolve, but at the same time, new welding methods applicable to advanced materials must be developed. For each newly developed material, the joining process must be re-assessed or developed to utilize the material effectively. The development of new materials requires specialized welding methods. Therefore, the welding of materials is constantly evolving, with new technologies and materials being introduced to the market.

As technology advances at an unprecedented pace, industries across all fields are experiencing significant changes in the way they operate. Welding is no exception, with exciting new trends emerging that are revolutionizing the way we join metals and other materials. Consequently, this Special Issue titled “Advances in Welding Process and Materials—2nd edition” contains contributions from several authors to the development and optimization of similar and dissimilar materials welding technologies so that the characteristics of welded joints meet current requirements related to quality, environmental protection, and reduction of energy and raw material consumption.

An important topic addressed in this volume is the analysis of the influence of different welding processes on the characteristics of welded joints in austenitic and duplex stainless steels.

In their paper, Tianqing Li and his collaborators [1] presented a review of the welding process for UNS S32750 super duplex stainless steel to provide useful information on welding this material.

UNS S32750 super duplex stainless steel is widely used in various leading industries, such as the marine, offshore oil and gas, or pulp and paper industries [1,2,3]. The mechanical properties and corrosion resistance of components made from this steel are influenced by the proportion of the two phases (austenite and ferrite) in the welded joint. A critical challenge in welding UNS S32750 is to maintain the correct proportion of the two phases in the welded joint. The two main methods providing solutions to balance these two phases are welding metallurgy control and optimization of welding parameters. It was found that high-energy-density welding processes, such as laser beam welding, electron beam welding, or plasma arc welding, are recommended to control the austenite/ferrite phase ratio in the UNS S32750 heat-affected zone [1]. At the same time, it must be considered that the cooling rate in laser and electron beam welding is higher compared to, for example, electric arc welding, a fact that affects the cooling separation of austenite from ferrite and, implicitly, the proportion of austenite in the welded joint. However, this can be remedied by applying appropriate post-weld heat treatments that can reduce ferrite content and improve welded joint performance.

Tianqing Li et al. [4] showed that nitrogen plays an important role in increasing the proportion of austenite in welded joints. Examining the effects of nitrogen on the microstructure and properties of SDSS 2507 welded joints obtained by gas-focused plasma arc welding (GS-PAW) showed that the addition of nitrogen increased the austenite content in the weld metal from 22.2% to 40.2% [4]. The study also highlighted an increase in the microhardness of austenite in the weld metal with the addition of nitrogen, due to the enrichment of the solid solution with nitrogen and the blocking of dislocation displacement.

In the same context of increasing the performance of duplex stainless steel welded joints, Xin Liu et al. [5] studied the influence of welding current on the corrosion resistance of heat-affected zones of HDR duplex stainless steel. HDR (high-chromium, duplex, corrosion-resistant) stainless steel shows higher corrosion resistance to seawater flow compared to copper alloys, which is why its use in ship applications is gradually expanding. The performance of components made by welding these steels depends on the behavior of the heat-affected zone (HAZ), because the welding process can generate the precipitation of harmful secondary phases or phase disproportion, leading to a decrease in corrosion resistance [6,7,8].

The corrosion behavior of the welding heat-affected zone of HDR duplex stainless steel welded using the TIG welding process was examined, and the experimental results show that a priming welding current of 70 A can promote the formation of austenite in the HAZ [5]. Increasing the covering welding current provides better resistance against pitting, but at the same time can lead to the precipitation of secondary phases at the grain boundary, which decreases resistance to intergranular corrosion. Therefore, setting the covering welding current to 100 A is a reasonable welding parameter [5].

Regarding the welding of austenitic stainless steels, such as AISI 316 type, numerous publications can be found [9,10,11,12,13,14]. However, there are a few publications that present a comprehensive comparison between microstructural and mechanical properties and the welding methods of AISI 316Ti steel [14]. In this context, it is noteworthy to mention that the study conducted by Piotr Noga and his collaborators on the influence of commonly used welding techniques, such as TIG and MIG, as well as high-energy EBW (electron beam welding) and PAW (plasma arc welding), on the microstructure and mechanical properties of 316Ti steel [9]. It was found that there are no defects or welding imperfections in any of the analyzed welding processes. From a microstructural point of view, the smallest amount of delta ferrite, approximately 2%, was found in welded joints obtained by the EBW method. The delta ferrite content in the structure of welded joints obtained by other methods (TIG, MIG, PAW) was slightly higher (approximately 4–5%) [9].

Mechanical tests carried out showed that the strength properties of joints welded by all the analyzed processes have similar values to the base material [9]. It was concluded that for welding using the PAW method, the joint efficiency was 93%, whereas for welding using the other methods, the joint efficiency was 98%. Hardness tests performed using the Vickers method revealed that the average hardness of welded joints, at the point of highest hardness (210 HV), is approximately 30 units higher compared to the base material (160 HV). The hardness values recorded in the heat-affected zone and in the axis of the welded joints are closely correlated with their microstructure, specifically with the delta ferrite content determined by metallographic analysis.

The performance of welded joints of austenitic stainless steels is influenced by both the welding method and process parameters. The welding process induces internal stresses in the welded joint, which, combined with the stresses during operation, may lead to cracks. An example is 316L stainless steel pipes used in the storage and transportation of low-temperature media, where tensile stress can occur near the heat-affected zone of the weld due to their low thermal conductivity and large coefficient of linear expansion [15,16]. Therefore, it is necessary that the residual stresses from the welding process be as low as possible.

To control crack defects caused by internal stress, Xiaowei Jiang et al. [17] simulated and studied the influence of welding process parameters on residual stress in the heat-affected zone in both the axial direction and around the circumference of the pipe using a finite element method. In their study, they constructed a thermal elastoplastic three-dimensional finite element (FE) model, and the simulation results were validated by measurements using a residual stress ultrasonic detector. It was found that the residual stress values obtained by the simulation method are quite close to the numerical values obtained by the experimental method, with an average deviation not exceeding 30 MPa [17].

It is worth noting that welding energy and welding speed are the main parameters that influence the transverse residual stress value but have almost no effect on the longitudinal residual stress value. It was determined that with a welding energy of 1007.4~859.3 J/mm and a welding speed of 6.6 mm/s, the maximum transverse residual stress is reached [17]. It was also found that different interlayer welding start positions influence the amplitude and distribution of the longitudinal residual stress of the pipe fitting, affecting the cyclic phase angle of the transverse distribution of residual stress in the seam center, but without changing its cyclic pattern.

The study of residual stress in welded joints is crucial for both similar joints and especially for dissimilar joints. In various industries such as automotive, aerospace, and naval, components made by welding different alloys are often used. Aluminum alloys, due to their light weight, are frequently used in applications in these fields [18]. Consequently, the study conducted by Zulqarnain Sarfaraz and his collaborators on residual stress in friction stir welding (FSW) of dissimilar aluminum alloys [19] is of interest from both scientific and practical perspectives. In their paper, they evaluated the influence of rotational and traverse speeds on the temperature distribution and resulting residual stress in the friction stir welding of dissimilar aluminum alloys AA2024–T3 and AA5086–O using a sequentially coupled thermomechanical 3D FEM model. They concluded that the two process parameters, i.e., rotation speed and traverse speed, affect the post-weld temperature distribution and demonstrated that temperatures remain higher on the retreating side (AA5086–O) than on the advancing side (AA2024–T3). These parameters also affect the longitudinal residual stress distribution. Simulation results validated by experimental tests showed that the rotational speed decreases the maximum residual stress, whereas increasing the traverse speed has the opposite effect [19].

Yang Hu et al. [20] also used the finite element method and provided technical guidance for welding aluminum alloy thin-walled, specially shaped tubes for bicycles. By analyzing the influence of TIG welding process parameters on the post-welding stress value, deformation, and temperature distribution, they succeeded in identifying the optimum welding parameters for 6061-T6 aluminum alloy: a welding current of 240 A, welding voltage of 20 V, and welding speed of 11 mm/s [20].

An important contribution regarding the joining of dissimilar materials was also made by Sajjad Arif and his collaborators [21]. They designed, developed, and tested machine learning models to estimate the ultimate tensile strength and hardness of a dissimilar AZ61 magnesium alloy/low carbon steel welded joint obtained by the FSW method. The results obtained, using an artificial neural network (ANN) model, were comparable to the experimental results, with a maximum error of 2.9% for hardness and 5.1% for ultimate tensile strength [21]. Therefore, synthetic prediction through machine learning modeling can be used for various FSW joints to optimize welding technology.

The finite element method was also used by Jianguang Yang et al. [22] to investigate the effects of different welding sequences on the temperature and the stress–strain fields during plasma welding of the shift fork shaft. A numerical model for two welding sequence schemes, unidirectional and bidirectional, was developed to optimize the plasma welding process of the shift fork shaft and minimize the adverse effects of residual stress. The simulation results show that reverse welding leads to a higher level of residual stress concentration [22,23]. Therefore, to obtain a high-quality weld, direct welding must be applied, and the optimal welding sequence must be used.

In many applications in the aerospace, marine, and transportation industries, advanced thermoplastic composites are being used more frequently due to their lightweight, high strength, and designability [24,25,26,27,28]. Therefore, Wanling Long and his team [24] conducted a study on improving the lap shear strength (LSS) of GF/PP (glass fiber-reinforced polypropylene) thermoplastic composites that were resistance welded by modifying stainless steel mesh heating elements (SSM) and using a silane coupling agent. Their study also investigated the influence of factors such as oxidation temperature, solvent properties, and solution pH through LSS testing. It was concluded that, compared to resistance-welded heads made of untreated SSM, those treated with an oxidation temperature of 500 °C, ethanol as solvent, and a solution pH of 11 show a 27.2% increase in tensile strength [24]. Therefore, the surface treatment under optimal conditions promotes increased resin infiltration into the SSM, thereby improving the weld strength (LSS) of resistance-welded joints [24].

An important contribution to this volume was made by Marius Bădicioiu and his colleagues [29] through their study on engineering applications of hardbanding technology in the petroleum industry. They focused on increasing drill pipe durability by establishing a hardbanding technology for reconditioning NC50 tool joints subjected to wear. Special equipment was designed and manufactured for applying high-quality hardbanding layers using the gas metal arc welding (GMAW) process and two different wear-resistant wires, ARNCO 100XT and FLUXOFIL M58 [29]. The experimental research performed under laboratory conditions allowed the optimization and validation of a reconditioning technology for NC50 tool joint boxes subjected to wear, obtaining hardbanding layer hardness values of 52–57 HRC [29]. Also, to ensure the reproducibility of the reconditioning technology, a welding procedure specification (WPS) was established.
